# Compensation Method for the Nonlinear Characteristics with Starting Error of a Piezoelectric Actuator in Open-Loop Controls Based on the DSPI Model

**DOI:** 10.3390/mi14040742

**Published:** 2023-03-27

**Authors:** Dong An, Ji Li, Songhua Li, Meng Shao, Weinan Wang, Chuan Wang, Yixiao Yang

**Affiliations:** 1 School of Mechanical Engineering, Shenyang Jianzhu University, Shenyang 110168, China; andong@sjzu.edu.cn (D.A.); liji@stu.sjzu.edu.cn (J.L.);; 2 School of Microelectronics, Fudan University, Shanghai 200433, China

**Keywords:** piezoelectric actuators, start-up accuracy, DSPI model, material physical characteristics

## Abstract

Nanopositioning stages with piezoelectric actuators have been widely used in fields such as precision mechanical engineering, but the nonlinear start-up accuracy problem under open-loop control has still not been solved, and more errors will accumulate, especially under open-loop control. This paper first analyzes the causes of the starting errors from both the physical properties of materials and voltages: the starting errors are affected by the material properties of piezoelectric ceramics, and the magnitude of the voltage determines the magnitude of the starting errors. Then, this paper adopts an image-only model of the data separated by a Prandtl-Ishlinskii model (DSPI) based on the classical Prandtl-Ishlinskii model (CPI), which can improve the positioning accuracy of the nanopositioning platform after separating the data based on the start-up error characteristics. This model can improve the positioning accuracy of the nanopositioning platform while solving the problem of nonlinear start-up errors under open-loop control. Finally, the DSPI inverse model is used for the feedforward compensation control of the platform, and the experimental results show that the DSPI model can solve the nonlinear start-up error problem existing under open-loop control. The DSPI model not only has higher modeling accuracy than the CPI model but also has better performance in terms of compensation results. The DSPI model improves the localization accuracy by 99.427% compared to the CPI model. When compared with another improved model, the localization accuracy is improved by 92.763%.

## 1. Introduction

Due to the inverse piezoelectric effect of piezoelectric material, the nanopositioning stage with piezoelectric ceramic material as the actuator exhibits advantages such as high accuracy, high resolution, and a short response time [1,2]. The effect is widely used in micro/nanofabrication, nanopositioning, high-speed electric spindles, atomic force microscopy, scanning tunneling microscopy, piezoelectric deformation mirrors, and other precision mechanical engineering fields [3,4,5,6,7,8]. However, the nonlinear output caused by the inherent properties of the piezoelectric material can affect the accuracy of the nanopositioning stage, requiring the effective compensation and control of the piezoelectric actuator [9,10]. The hysteresis characteristic is the most important factor contributing to the nonlinear characteristics [11,12].

In order to improve the positioning accuracy of the nanopositioning stages, the hysteresis characteristics of piezoelectric actuators have been extensively studied by several international scholars [13,14,15,16,17]. Song et al. proposed a novel and improved Preisach model to identify and model the hysteresis observed in piezoelectric actuators, and the improved method can handle the frequency dependence by employing a time-derivative correction technique to keep the deviation in a low percentage range [18]. Lin et al. reconstructed the Bouc-Wen model, the Dahl model, and the Duhem model into the generalized Duhem model to compare the performance of various hysteresis models relative to the tracking reference. Since the Duhem model includes both electrical and mechanical domains, it has a lower modeling error compared to the other two hysteresis models [19]. Wang et al. proposed a novel and improved Bouc-wen (MBW) model to describe the asymmetric hysteresis of piezoelectric actuators using polynomial-based nonhysteresis components to achieve asymmetric hysteresis characteristics [20]. Liu et al. proposed a Hammerstein model based on fractional-order rate correlation. The improved Bouc-wen model is used to describe the asymmetric hysteresis characteristics of PEA, and the fractional order model is used to describe the dynamic characteristics of PEA. The nonlinear rate-dependent hysteresis model can describe the dynamic characteristics of PEA more accurately [21]. Wang et al. investigated the effect of drive voltage amplitude and frequency on the hysteresis characteristics of a single-axis piezoelectric actuator in a piezoelectric displacement table and proposed the use of a fitting factor R2 to evaluate the hysteresis compensation accuracy of the MPI model [22].

In particular, the Prandtl-Ishlinskii model has a wide range of applications [23,24]. The classical Prandtl-Ishlinskii (PI) model is an image-only class of operator models. The PI model is formed by the weighted superposition of Play operators. Although the Play operator can describe the hysteresis properties well, its symmetry limits the modeling accuracy of PI models [25,26]. Improved classical PI models using methods such as adding asymmetric terms, generalized models, segment fitting, or adding parameters to the model can enhance the compensation accuracy to some extent [27,28,29].

Although many domestic and overseas scholars have been working on various methods to compensate and for and control the nonlinear characteristics of piezoelectric actuators, they have not taken into account the effect of starting errors on the hysteresis characteristics. Open-loop control is used in many scenarios, such as the error compensation of the ceramic ball bearing rotor system and tooth profile motors [30], where neglecting the effect of the starting errors can reduce the accuracy of the compensation. Therefore, it is crucial to propose a new compensation method to achieve the required accuracy for compensating nonlinear start-up error characteristics under the open-loop control of a piezoelectric actuator.

In order to address the problem of the nonlinear starting errors of piezoelectric actuators under open-loop control, this paper proposes a scheme to improve the open-loop starting accuracy of the data separated by the Prandtl-Ishlinskii model (DSPI). The causes of starting errors were analyzed in terms of both physical material properties and voltage, and in the process, two starting error characteristics were identified. The DSPI model is modeled based on start-up error characteristic 3. The results show that the DSPI model can describe the system with high precision and can solve the problem of nonlinear start-up errors that exist under open-loop control.

The framework of this paper is as follows. The second part introduces a designed nanopositioning stage with a dual parallel guidance mechanism and introduces the experimental system. In the third section, the influencing factors that may lead to the generation of start-up errors are analyzed. The fourth section analyzes the hysteresis characteristics and derives a DSPI model that is more suitable for fitting the nonlinear start-up error characteristics under open-loop control. The control method and the DSPI inverse model are given. The fifth section describes the modeling results of the DSPI model and gives the control comparison and validation experiments between the DSPI model and the traditional PI model. The final sixth section is the conclusion of this paper.

## 2. Background

This section is divided into two parts. The first part describes a designed nanopositioning stage with a dual parallel guidance mechanism. The second part describes the experimental system which is used to conduct the voltage-displacement experiments.

### 2.1. Mechanical Design

**Figure 1:**
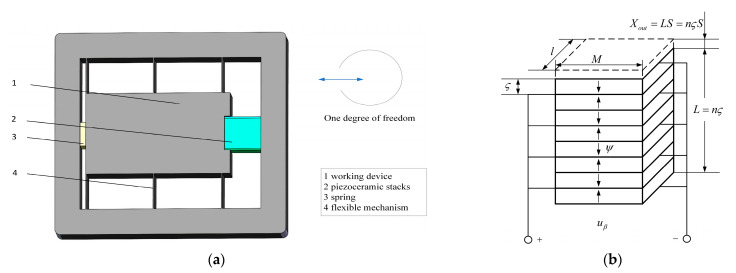
Three-dimensional schematic: (**a**) one-degree-of-freedom nanopositioning platform; (**b**) piezoelectric ceramic stacking.

Figure 1 presents a three-dimensional schematic of the nanopositioning stage, which achieves high-precision motion using a fully flexible mechanism. The flexible mechanism has a nanometer range of elastic deformation motion, and it is able to ignore friction to achieve motion and force transfer. Considering the small thickness of the flexible hinges, wire-cut machining is used for the material. Therefore, the cutout of the flexible hinge is chosen to be more easily machined with a rectangular profile flexible hinge. As a basic component of the flexible guiding mechanism, the flexible hinge is an important part of the nanopositioning platform [31]. The moving part of the nanopositioning stage is fixed to the surrounding rigid frame mechanism by the flexible hinge, and the motion characteristics of the moving part are completely dependent on the deformation of the flexible hinge. In order to strictly ensure the accuracy of the positioning stage, the slow-walking wire-cut processing technology with micron-level accuracy was adopted. At the same time, materials with a low coefficient of thermal expansion were selected.

As shown in Figure 1a, the stage is a direct drive nanopositioning stage with one degree of freedom, and the displacement is output by a double parallel flexible hinge guidance mechanism. The displacement output of the direct drive nanopositioning stage is similar to the displacement output of the piezoelectric ceramic actuator, and it can clearly present the properties of the material itself. The selected nanopositioning platform was obtained from Sanying MotionControl Instruments, Ltd of Tianjin, China. The platform’s external dimensions are 120 mm × 150 mm × 30 mm, the material is aluminum alloy 7050-T7451, and the material elastic modulus is 72 Gpa.

The selected piezoelectric ceramic actuator is a stacked piezoelectric ceramic actuator. It has a maximum displacement of 50 μm and an operating voltage of −30 V to 180 V. As shown in Figure 1b, ς is the thickness of a single piezoelectric ceramic chip. ψ is the direction of polarization. l is the width of the piezoelectric ceramic sheet (m). M is the length of the piezoelectric ceramic sheet (m). n is the number of layers of piezoelectric stacked ceramic sheets. *L* is the total height of the piezoelectric stack (m). S is the strain in the longitudinal direction of each piezoelectric ceramic. $u_{\beta }$ is the driving voltage of the piezoelectric stack (V). $X_{out}$ is the total output displacement in the longitudinal direction of the piezoelectric stack (m).

### 2.2. Experimental Equipment

**Figure 2:**
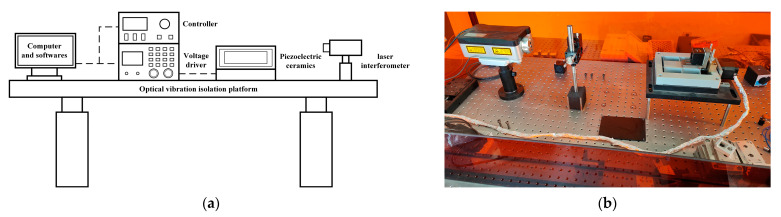
Nano platform experimental system: (**a**) system schematic diagram; (**b**) system build diagram.

The experimental system was built using a positioning stage, a laser interferometer, a reflector, a controller, and a computer with the controller operating software, as shown in Figure 2a. The laser interferometer chosen is the Renishaw XL-80 series, which achieves ±0.5 ppm accuracy. It can achieve 1 nm resolution in a good experimental environment. The power supply driver was the HVA-150D.A3 model from Harbin Core Tomorrow Company, and the input voltage is variable from 0 to 150 V. Figure 2b shows the experimental system built for the experiment.

In this paper, the output voltage-displacement characteristics of the one-degree-of-freedom direct drive nanopositioning stage were tested by the nanopositioning experimental system that is shown in Figure 2. The data collected from the experiments were simulated and analyzed using MATLAB software to build a DSPI model.

## 3. Causes of Starting Error

This section describes the causes of starting errors in two parts. The first part analyzes the effect of the physical properties of materials on the starting error. The second part analyzes the effect of the magnitude of voltage on the starting error.

### 3.1. Start-Up Error Characteristic 1

Piezoelectric ceramics [32] possess the ability to undergo an electrostrictive effect when exposed to an electric field due to the dielectric stretching effect caused by dielectric polarization. In the presence of an electric field, the dielectric molecules are polarized, resulting in dielectric stress and corresponding deformation. Due to the strong mutual attraction between the nucleus and the electron, the applied electric field is not sufficient to destroy the dielectric properties. Therefore, it is concluded that the electrostriction coefficient is much smaller in order of magnitude than the inverse piezoelectric effect.

In general, the inverse piezoelectric effect can be expressed as:

(1) Ρ=dE

The electrostrictive deformation is proportional to the square of the electric field strength, independent of the electric field polarity. Based on Equation (1), the piezoelectric ceramic displacement of the electrostriction effect is analyzed concerning the electric field as:

(2) $$P=\mu E^{2}+dE$$

In Equation (2), P is the axial displacement in the direction of piezoelectric ceramic energization, d is the inverse piezoelectric effect coefficient, μ is the electrostriction effect coefficient, and E is the electric field strength. In terms of macroscopic performance, the electrostriction effect is extremely weak, and its resulting output displacement of the piezoelectric ceramic is usually neglected.

**Figure 3:**
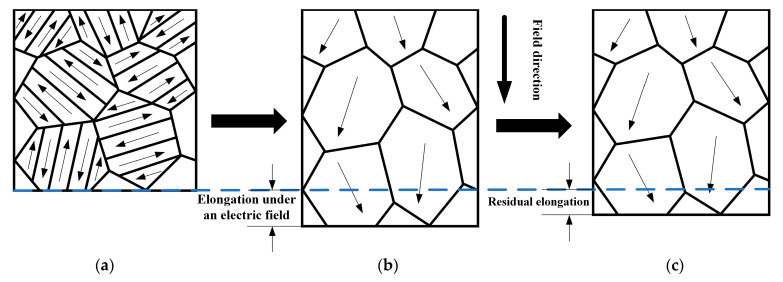
Schematic diagram of the change of electric domains in piezoelectric ceramics during the polarization process: (**a**) before polarization; (**b**) during polarization; (**c**) after polarization.

Synthetic piezoelectric ceramics originally have no piezoelectricity and are polarized to have a piezoelectric effect. When microscopically explained, the displacement changes in piezoelectric ceramics are caused by electric dipole polarization. The electric domain is the average effect of electric dipole distance after the interaction between electric dipoles, which occurs spontaneously by sintering. In the steady-state action, the arrangement of electric domains is irregular when the piezoelectric ceramics have no piezoelectric effect. After polarization operation, that is, when an electric field is applied to the piezoelectric ceramic and the field strength exceeds a certain value, if the direction of the electric field is the same as the polarization direction, the electric domains inside the piezoelectric ceramic will flip and elongate, and the domain boundaries will also be deformed by extension. For the grains, the flip of the non-180° electric domains has a significant effect on the boundary displacement [33]. After temperature and electric field removal, the electric dipole within the crystal does not return to its initial state, and residual polarization intensity exists. The process of the electric domain change during polarization is depicted in Figure 3.

After the external field is applied to the piezoelectric ceramic actuator and then removed, there will be a residual elongation compared to that before the external field is applied. This means that when the piezoelectric ceramic actuator is given a voltage from 0 V to 150 V and then to 0 V, the initial 0 V elongation of the piezoelectric ceramic actuator is 0 μm, and the end 0 V elongation of the piezoelectric ceramic actuator is greater than 0 μm. This residual elongation results in the displacement value failing to return to the 0 point, leading to a subsequent start-up error.

This gives the first characteristic of the start-up error: when the given voltage drops from the maximum value to 0 V, its displacement value is not 0 (it cannot return to the origin) and the start-up error is generated by starting the 1st turn of data.

### 3.2. Start-Up Error Characteristic 2

Through several experimental data collection studies, it is found that the starting error is also influenced by the voltage. When the given voltage becomes larger, the starting error will become larger; when the given voltage becomes smaller, the starting error will become smaller.

**Figure 4:**
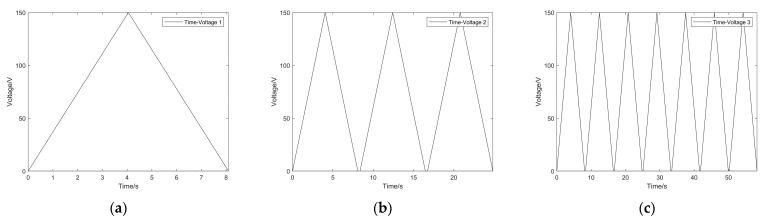
Experimental loading voltage: (**a**) first cycle voltage after start-up; (**b**) the first three cycles of voltage; (**c**) the first seven cycles of voltage.

As shown in Figure 2, the output voltage-displacement characteristics of a one-degree-of-freedom direct-drive nanopositioning stage are tested. The data is collected by adjusting the laser interferometer, computer, and controller. The initial drive voltage of the piezoelectric ceramic actuator is 0 V. A linear voltage rise signal is given from 0 V to 150 V. The displacement values are measured and recorded by laser interferometer every 10 V. A linear voltage is given immediately afterward to reduce the signal from 150 V to 0 V. The displacement values are measured and recorded by laser interferometer every 10 V. The data were collected throughout the experiment by performing multiple measurements. As shown in Figure 4a, it is the 1st cycle voltage after start-up. As shown in Figure 4b, it is the first 3 cycles of voltage after starting. As shown in Figure 4c, it is the first 7 cycles of voltage after starting.

**Figure 5:**
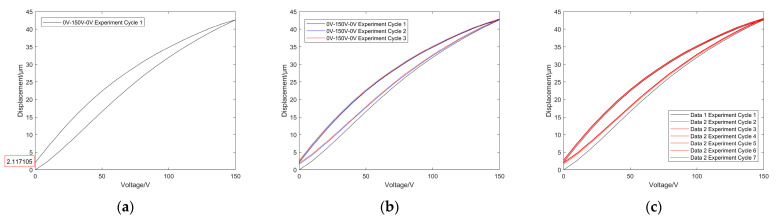
Experimental voltage-displacement curves: (**a**) voltage-displacement curve of the first cycle after start-up; (**b**) voltage-displacement curve of the first three cycles; (**c**) voltage-displacement curve of the first seven cycles.

The experiments conducted provide three sets of data, which include the voltage-displacement curve of the first cycle of voltage after start-up (Figure 5a). The horizontal axis of this plot depicts the voltage, whereas the vertical axis represents the microdisplacement of the stage output. The voltage-displacement curve of the first three cycles of voltage is shown in Figure 5b. The voltage-displacement curve for the first seven cycles of voltage is shown in Figure 5c. It is crucial to note that the data for cycles 2, 3, 4, 5, 6, and 7 are not within the confines of the data of cycle 1.

The desired voltage-displacement curve should be linear. By analyzing the experimental data, it was clearly observed that the experimental curves showed nonlinearity. The voltage-displacement curve at linear voltage has hysteresis characteristics. Therefore, an effective compensation method is needed to address the hysteresis effect of piezoelectric ceramic characteristics on the control at linear voltages.

**Figure 6:**
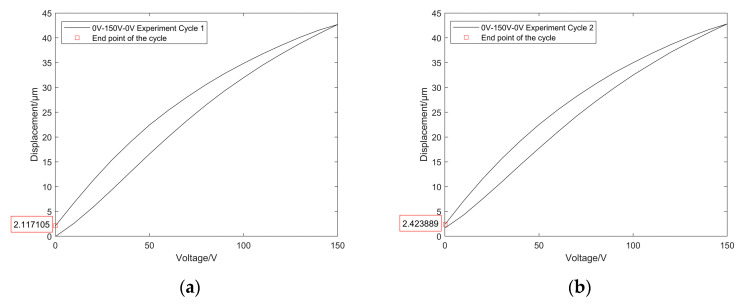
Start-up error diagram: (**a**) start-up first cycle error; (**b**) the second cycle.

The displacement at the initial 0 V voltage of a cycle is defined as $d_{\alpha 1}$ The displacement at 0 V at the end of a cycle is defined as $d_{\alpha 2}$ Then, the error is $\Delta_{\alpha }=d_{\alpha 1}-d_{\alpha 2}$. As shown in Figure 6, a significant start-up error can be observed in the voltage-displacement curve of the experimental data of the first cycle after start-up.

Three sets of test experiments at different voltages were tested. The initial drive voltage of the piezoelectric ceramic actuator is 0 V. The linear voltages of 0–150–0 V, 0–100–0 V, and 0–50–0 V are given, respectively. As shown in Table 1, the start-up error values for the three sets of test experiments are shown.

By observing the test results, it is apparent that there was a considerable deviation in the start-up error value of the first cycle data after a start-up of 2.117105 μm at 0–150–0 V. However, the start-up error value at 0–100–0 V was much lower, with only 1.411403 μm observed in the first cycle of data after start-up. Even lower still was a start-up error value of only 0.827996 μm observed at 0–50–0 V, which was significantly less than the start-up error value observed at 0–100–0 V.

The above experiments can be used to obtain characteristic 2 of the starting error: the magnitude of the voltage will have an effect on the magnitude of the starting error. When the maximum value of the given voltage is larger, the starting error will be larger. When the maximum value of the given voltage is smaller, the starting error will be smaller.

The Section 3.2.1 analyzes the reasons why voltage magnitude can have an impact on the magnitude of the starting error.

#### 3.2.1. Causes of Voltage-Affected Starting Error

**Figure 7:**
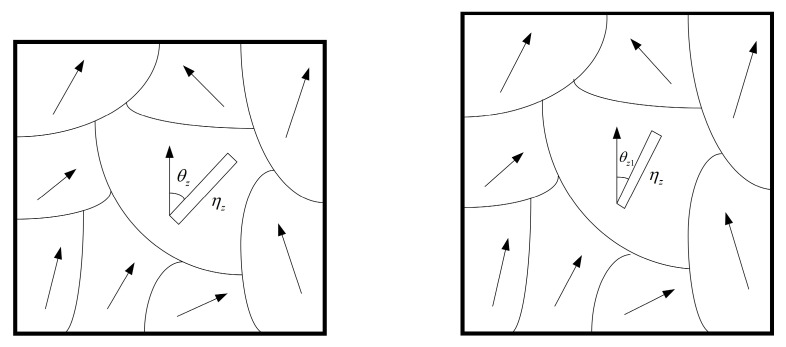
Schematic diagram of electric domain steering by electric field polarization.

The length of the zth domain is set to $\eta_{z}$ and the angle between the spontaneous polarization direction of the domain and the electric field direction is set to $\theta_{z}$ If an electric field is applied externally, the electric domain is deflected by the electric field, and the deflection direction is close to the electric field direction. At this time, the angle between the spontaneous polarization direction of the electric domain and the electric field direction changes from $\theta_{z}$ to $\theta_{z1}$, as shown in Figure 7.

As a result, the relation for the amount of change in deformation $\Delta L_{z}$ of a single electric domain under the action of an external electric field is obtained:

(3) $$\Delta L_{z}=\eta_{z}\cos\theta_{z1}-\eta_{z}\cos\theta_{z}=\eta_{z}\left(\cos\theta_{z1}-\cos\theta_{z}\right)$$

Then, the macroscopic displacement is the sum of all the electric domain form variables:

(4) $$\Delta L=\sum \Delta L_{z}=\sum \eta_{z}\left(\cos\theta_{z1}-\cos\theta_{z}\right)$$

Both $\eta_{z}$ and $\theta_{z}$ are constants. $\theta_{z1}$ varies with the field strength of the external electric field. Then, Equation (4) can be Taylor expanded at the constant $\theta_{z}$ and retained to the third order, as shown in Equation (5):

(5) $$\Delta L=\sum \eta_{z}\left[-\left(\theta_{z1}-\theta_{z}\right)\sin\theta_{z}-\frac{\cos\theta_{z}}{2!}{\left(\theta_{z1}-\theta_{z}\right)}^{2}+\frac{\sin\theta_{z}}{3!}{\left(\theta_{z1}-\theta_{z}\right)}^{3}\right]$$

As shown in Equation (5) above, as $\left(\theta_{z1}-\theta_{z}\right)$ becomes larger, the output displacement ΔL of the piezoelectric ceramic actuator due to the non-180° domain steering becomes larger. From the start-up error characteristic 1 of Section 3.1, it can be seen that when the external field action acts on the piezoelectric ceramic actuator and then removes it, there will be a residual elongation compared to the amount before the external field action. When the voltage applied to the piezoelectric ceramic actuator becomes larger, $\left(\theta_{z1}-\theta_{z}\right)$ becomes larger. When the given voltage drops from 150 V to 0 V, the larger the resulting starting error becomes; conversely, when the voltage applied by the piezoelectric ceramic actuator becomes smaller, $\left(\theta_{z1}-\theta_{z}\right)$ becomes smaller. When the given voltage decreases from 150 V to 0 V, the resulting starting error becomes smaller.

Start-up accuracy can have a significant impact on the accuracy after hysteresis compensation, especially in open-loop control, where more errors are accumulated. It is extremely important to analyze the causes of the starting errors and, at the same time, adopt an effective method to achieve compensation for the nonlinear starting error characteristics under the open-loop control of the piezoelectric actuator. Therefore, this paper will propose a DSPI model capable of eliminating start-up errors while compensating for nonlinear start-up error characteristics of piezoelectric actuators under open-loop control.

## 4. Modeling

This section describes the process of building the DSPI model in four parts. In the first part, the CPI model and its inverse model are developed, and the curves describing the hysteresis characteristics of the CPI model are obtained. The second part elaborates on the start-up error characteristic 3 found during the modeling of the improved CPI model. Then, the basis for the DSPI model modeling is illustrated. In the third part, the DSPI model is developed, and the description curve of the DSPI model is given. In the fourth section, the control method is introduced. the description curve of the DSPI inverse model is built.

### 4.1. CPI Model

**Figure 8:**
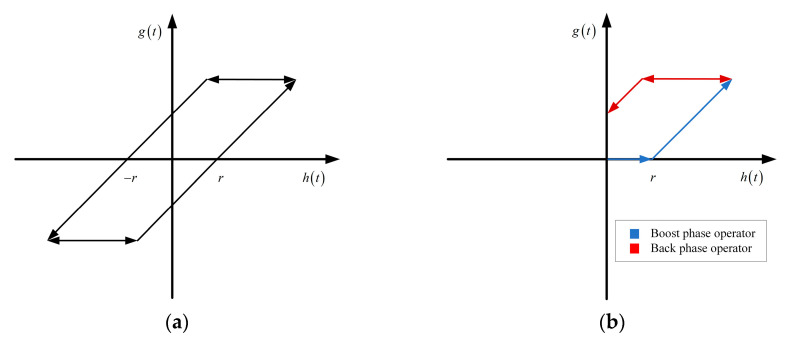
Play operator: (**a**) the complete operator model; (**b**) the one-sided operator model.

The traditional PI model (CPI model) is a weighted superposition of a finite number of play operators with different thresholds. The play operator with slope 1 is shown in Figure 8a. When the input signal is h(t), the play operator expression $g\left(t_{n}\right)$ can be expressed as

(6) $$\left\{ \begin{array}{l} g\left(0\right)=\max\left\{h\left(0\right)-r,\min\left[h\left(0\right)+r,g_{0}\right]\right\}\\ g\left(t_{n}\right)=\max\left\{h\left(t_{n}\right)-r,\min\left[h\left(t_{n}\right)+r,g_{n-1}\right]\right\} \end{array}\right.$$

In Equation (6), *r* is the threshold value of the play operator, n∈[1,e], $0=t_{0}<t_{1}<t_{2}<\cdots <t_{s}$ is the division on the input signal interval. When using the CPI model to study the piezoelectric effect hysteresis problem, the initial form variable of the displacement is usually noted as 0, so $g_{0}=0$ is taken.

The play operator has a high similarity in appearance to the hysteresis curve of piezoelectric ceramics. The CPI model describing piezoelectric hysteresis can be obtained by superimposing a finite number of play operators weighted by each of the above output characteristics. Equation (7) can be expressed as

(7) $$G\left[h\left(t\right)\right]=\omega_{0}\cdot h\left(t\right)+\sum_{i=1}^{n}\omega_{i}\cdot g_{i}\left(t\right)$$

In Equation (7), G[h(t)] is the output of the CPI model corresponding to the operator input h(t). $\omega_{0}$ is the first weight, which is usually taken as a positive number. The ith operator corresponds to the output $g_{i}\left(t\right)$ with the corresponding threshold $r_{i}$ and the corresponding weight $\omega_{i}$.

**Figure 9:**
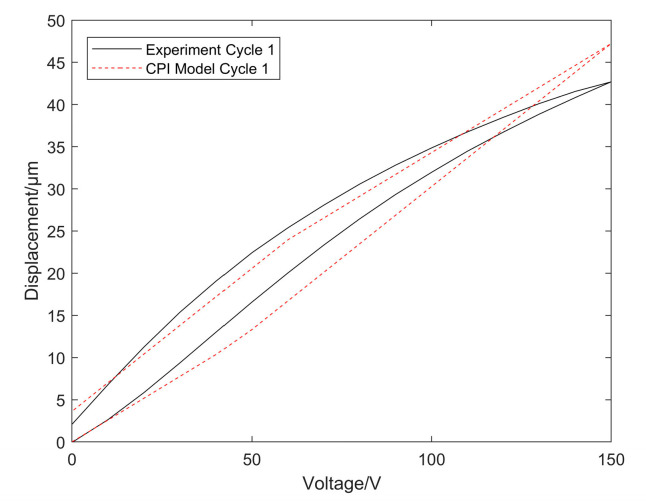
CPI model modeling—the first cycle of experimental data after start-up.

The CPI model modeling of the experimental data for the first lap after startup is shown in Figure 9. The modeling results show that the hysteresis characteristics are not similar to the first cycle experimental curve, and the compensation of the nonlinear start-up error characteristics of the open-loop control of the piezoelectric actuator cannot be achieved. Therefore, it is necessary to enhance the CPI model to achieve higher accuracy in fitting the data.

In the process of model improvement, the start-up error characteristic 3 was identified. The Start-up error characteristic 3 is described in Section 4.2 below. Additionally, the basis of the DSPI model modeling is illustrated.

### 4.2. Start-Up Error Characteristic 3

Through the analysis in Section 3.1 of this paper, the start-up error characteristic 1 was obtained: “Start-up first cycle data when the given linear voltage drops from 150 V to 0 V when the displacement value is not 0 and does not return to the origin; this will generate the start-up error”. Based on this characteristic, the experimental data were analyzed and it was noted that the startup error was more significant in the first cycle data. Therefore, it cannot be modeled with the same model as the data in cycles 2,3,4,5,6,7…; otherwise, it will lead to a larger modeling error, and the accuracy of the model will be lower. Therefore, this paper intends to model the first start-up cycle data separately. The start-up error $\Delta_{\alpha }$ of the first cycle data is set to 0. In the process of modeling, this paper finds that, after the start-up error $\Delta_{\alpha }$ of the first cycle is set to 0, the modeling starts again with ‘0’, and the errors of the data after the 2,3,4,5,6,7 … cycles are small.

Specifically for the data after the 2,3,4,5,6,7 … cycles, it was found that the error after starting from the second cycle was only nearly one-fifth of a percent of the first cycle start error. The error after the 3,4,5,6,7th… cycle data also decreases and tends to zero compared to the error in the second cycle. Since the deviation is small, it is negligible. This phenomenon is attributed to start-up error characteristic 3: the error from the second turn onward is so small that it is negligible. Therefore, it is reasonable to consider only the effect of the first turn of the start-up error. It is important to point out that the 2,3,4,5,6,7 … cycles are not the inner loops of the first cycle.

Start-up error characteristic 3 and start-up error characteristic 1 determine that the classical PI model can be successfully improved to achieve the compensation of the nonlinear start-up error characteristics of the piezoelectric actuator under open-loop control.

Since the first cycle data have a start-up error, the data after the 2,3,4,5,6,7 … cycles have high repeatability and no bias. Based on this characteristic mentioned above, we can model the data in two parts: modeling the data of the first cycle separately and modeling the data after the 2,3,4,5,6,7 … cycles separately. The advantage of this modeling is that the start-up error produced by the first cycle data does not affect the model accuracy of the subsequent 2,3,4,5,6,7… cycle data. A schematic diagram of the experimental data divided into two parts is shown in Figure 5c. The first cycle data of the black line is the first part, and the 2,3,4,5,6,7… cycles data of the red line is the second part.

### 4.3. Data-Separated Prandtl-Ishlinskii Model

A data-separated Prandtl-Ishlinskii Model is proposed based on the description of the modeling basis of the DSPI model in Section 4.2.

The DSPI model is modeled in two parts: for the first cycle data and the data after the 2,3,4,5,6,7 … cycles, these are modeled separately.

The first cycle data were used as the new first cycle. Since the 2,3,4,5,6,7 … cycle data have high repeatability and no bias, the 2,3,4,5,6,7 … cycle data were averaged as new cycle 2.

Suppose the displacement value of the data collected in the first cycle at 0–150–0 V is a_1_, a_2_, a_3_ … a_x_. The displacement value of the data collected in the second cycle is b_1_, b_2_, b_3_ … b_x_. The displacement value of the data collected in the third cycle is c_1_, c_2_, c_3_ … c_x_. The displacement value of the data collected in the nth cycle is n_1_, n_2_, n_3_ … n_x_.

**Figure 10:**
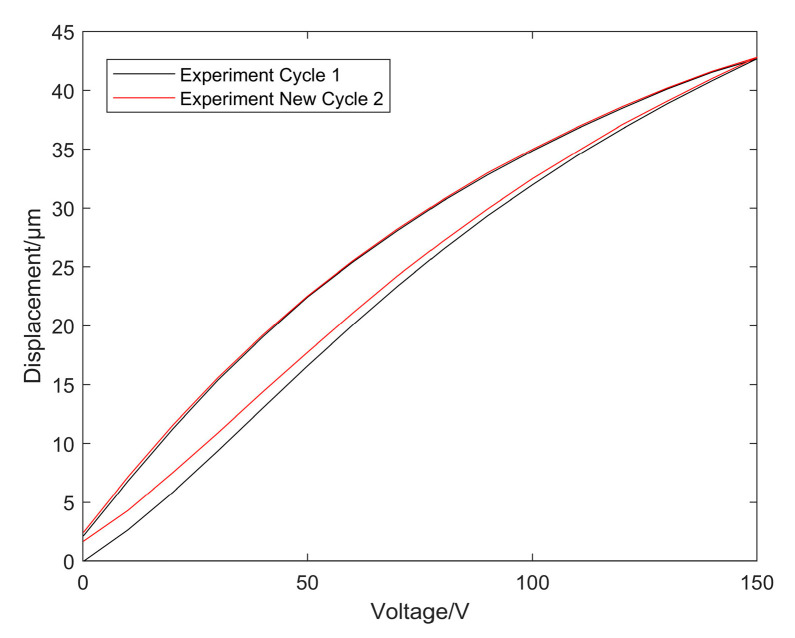
Voltage-displacement curves for the first cycle and the new second cycle.

The corresponding displacement values of the 2,3,4,5,6,7 … nth cycles are summed, and the average value is taken: (b_1_ + c_1_ +…+ n_1_)/(n − 1), (b_2_ + c_2_ +…+ n_2_)/(n − 1), (b_3_ + c_3_ +…+ n_3_)/(n − 1), … (b_n_ + c_n_ +…+ n_n_)/(n − 1). After averaging, the new displacement value of the second cycle is d_1_, d_2_, d_3_ … d_x_. As shown in Figure 10, the voltage-displacement curves of the first cycle data and the new second cycle data obtained from the data reclassification process are presented.

The first cycle data are modeled in two parts. The first part is to model the data separately at a given voltage from 0 V to 150 V. Equation (8) can be expressed as

(8) $$G\left[h\left(t\right)\right]=\omega_{0}\cdot h\left(t\right)+\sum_{i=1}^{n}\omega_{i}\cdot g_{i}\left(t\right)$$

The second part is to model the data separately at a given voltage of 150–0 V. Equation (9) can be expressed as

(9) $$G\left[h\left(t\right)\right]=\Delta_{\alpha }+\omega_{0}\cdot h\left(t\right)+\sum_{i=1}^{n}\omega_{i}\cdot g_{i}\left(t\right)$$

In Equations (8) and (9), $\Delta_{\alpha }$ is the deviation and G[h(t)] is the output of the CPI model corresponding to the operator input h(t). $\omega_{0}$ is the weight of the first term, which is usually a positive number. The ith operator corresponds to the output $g_{i}\left(t\right)$. It has a corresponding threshold $r_{i}$ and a corresponding weight $\omega_{i}$.

The equation of the new second cycle is Equation (9).

**Figure 11:**
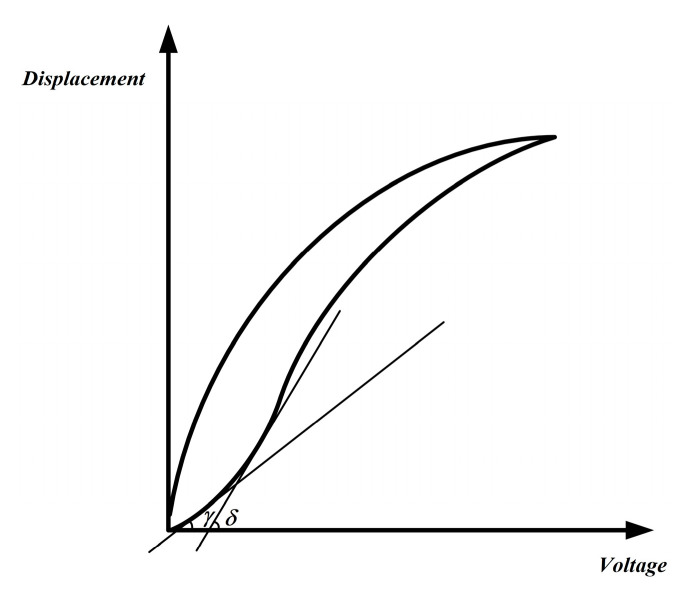
Schematic diagram of the tangent to the voltage-displacement hysteresis curve.

The hysteresis rate corresponding to a voltage position on the voltage-displacement curve is obtained by superimposing the respective weights of the play operators in the PI model at that point. The weight $w_{i}$ of the ith operator depends on the angle γ between the tangent line of the hysteresis curve at that point and the voltage-axis. As shown in Figure 11, the angle corresponding to the tangent line at each point on the hysteresis loop is different.

The voltage-displacement data can approximate the characteristic curve and, thus, establish the voltage-displacement co-ordinate system. If m is the mth data obtained from the experiment, the hysteresis loop passes through the point $\left(v_{m},d_{m}\right)$. Define the equation of the tangent line $y_{\tan}\left(v\right)$ of the hysteresis rate at  as

(10) d=s(v)⋅v+b(v)

In Equation (10), s(v) is the tangent slope of the hysteresis rate at v. b(v) is the tangent intercept of the hysteresis rate at v.

The slope of the hysteresis tangent line reflects the trend of hysteresis at that point. The slope of the hysteresis tangent line s(v) can be expressed as

(11) $$s\left(v\right)=\frac{\Delta d}{\Delta v}=\frac{d_{m+1}-d_{m}}{v_{m+1}-v_{m}}$$

In Equation (11), $\left(v_{m},d_{m}\right)$ and $\left(v_{m+1},d_{m+1}\right)$ are adjacent data and conform to $\min(v_{m},v_{m+1})\leq v\;<\;\max\left(v_{m},v_{m+1}\right)$.

**Figure 12:**
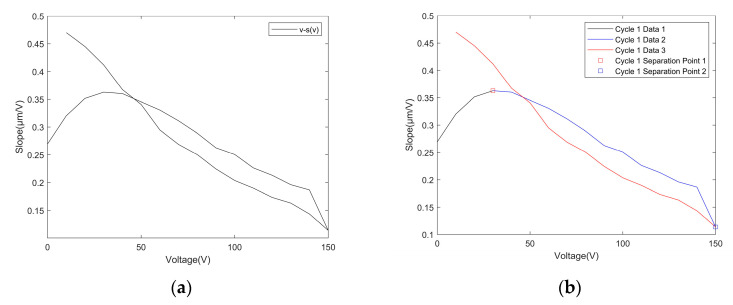
Voltage–hysteresis tangent slope plots: (**a**) cycle 1 v-s(v) plot; (**b**) cycle 1 slope data separation plot.

**Figure 13:**
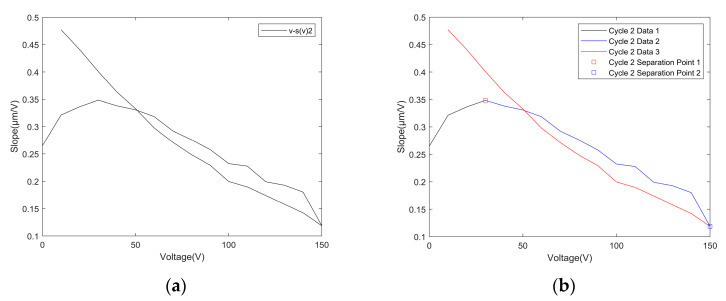
Voltage–hysteresis tangent slope plots: (**a**) new second cycle v-s(v) plot; (**b**) new second cycle slope data separation plot.

The voltage-hysteresis tangent slope diagram can visually characterize the hysteresis variation with voltage. Different input voltages cause different patterns of hysteresis tangents. Figure 12a and Figure 13a are the v−s(v) plots of the experimental data for cycle 1 and the new cycle 2.

Figure 12 and Figure 13 illustrate the relationship between different voltages and corresponding slopes, which can either display an upward or a downward trend. In Figure 12b, Cycle 1 Separation Point 1 and Cycle 1 Separation Point 2 are the inflection points of the slope change of the voltage-hysteresis tangent line for the first cycle data. This inflection point is used as the separation point for the data separation process. In Figure 13b, Cycle 2 Separation Point 1 and Cycle 2 Separation Point 2 are the inflection points of the slope change of the voltage-hysteresis tangent line for the new second cycle data. This inflection point is used as the separation point for the data separation process.

### 4.4. Compensated Control and DSPI Inverse Model

The DSPI model obtains an accurate approximation of the voltage-displacement correspondence by describing the nonlinear start-up error characteristic curve. The feedforward control method system is one of the common compensating control methods used in engineering [34,35]. The compensation control results can be corrected for changes in the disturbances or given values by using precollected experimental data information and applying the precontrol method at the front end of the system. In order to achieve accurate compensation for the start-up error characteristic curve, the displacement-voltage correspondence of the DSPI inverse model is used as the feedforward control.

**Figure 14:**
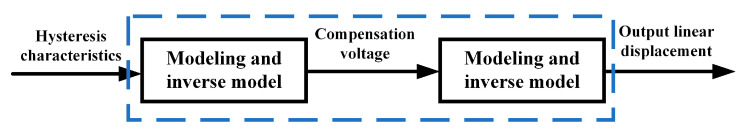
Feedforward compensation control schematic.

The PI model used in the modeling of the DSPI model is a phenomenological model. It directly describes the start-up error characteristics without explaining the intrinsic causes. Therefore, the feedforward control is the most effective method to compensate for the nonlinear start-up error characteristics under open-loop control. The feedforward compensation control diagram is displayed in Figure 14.

The inverse model of the DSPI model is equivalent to the recombination of the PI model after the data separation of the components. Therefore, the DSPI inverse model equation is consistent with the PI inverse model. Equation (12) can be expressed as

(12) $$G^{-1}\left[g\left(t\right)\right]=\omega_{0}^{-1}\cdot g\left(t\right)+\sum_{i=1}^{n}\omega_{i}^{-1}\cdot h_{i}\left(t\right)$$

In Equation (12), $G^{-1}\left[g\left(t\right)\right]$ is the output corresponding to the input g(t) of the CPI inverse model operator. $h_{i}\left(t\right)$ is the output of the ith operator, so it corresponds to the positive CPI model, $h_{0}=0$. The threshold $r_{i}^{-1}$ and the weight coefficient $\omega_{i}^{-1}$ of the CPI inverse model can be expressed as

(13) $$\left\{ \begin{array}{l} r_{i}^{-1}=\sum_{j=1}^{i}\omega_{j}\cdot \left(r_{i}-r_{j}\right)\\ \omega_{0}^{-1}=\frac{1}{\omega_{0}}\\ \omega_{i}^{-1}=-\frac{\omega_{i}}{\left(\omega_{0}+\sum_{j=1}^{i-1}\omega_{j}\right)\left(\omega_{0}+\sum_{j=1}^{i}\omega_{j}\right)} \end{array}\right.$$

## 5. Experiments and Discussion

This section is the experimental results and discussion section of this paper. In Section 4, the DSPI model was proposed to better describe the nonlinear start-up error characteristic curve under the open-loop control of the piezoelectric actuator. In the first part of this section, the descriptive curves of the DSPI model are given. In the second part, the descriptive curves of the DSPI inverse model are given. The compensated control voltage obtained from the DSPI inverse model is verified in Part III and Part IV. The error of its inverse model is also analyzed.

### 5.1. Results of the DSPI Model

**Figure 15:**
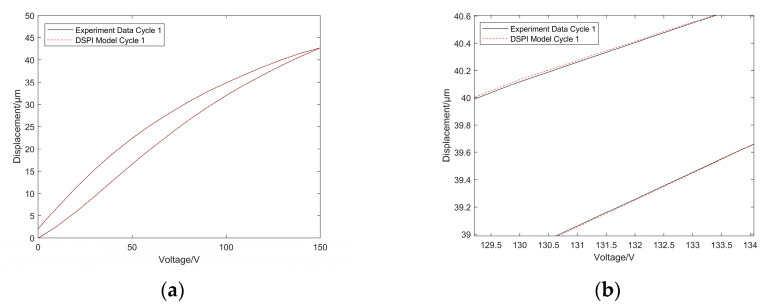
DSPI model: (**a**) modeling of the first cycle data hysteresis characteristics; (**b**) local enlargement.

As shown in Figure 12b, there are two separation points: Cycle 1 Separation Point 1 and Cycle 1 Separation Point 2, which separate the data into three parts. The one-sided play operator is selected for each part of the data by condition. The data of each part of the first cycle separation was refitted, as shown in Figure 15a. The DSPI model has good articulation at the separation point, its model accuracy at the start is high, and the overall model fit is very good. Moreover, the hysteresis characteristics of the first cycle data have been partially enlarged and modeled, which can be observed in Figure 15b.

**Figure 16:**
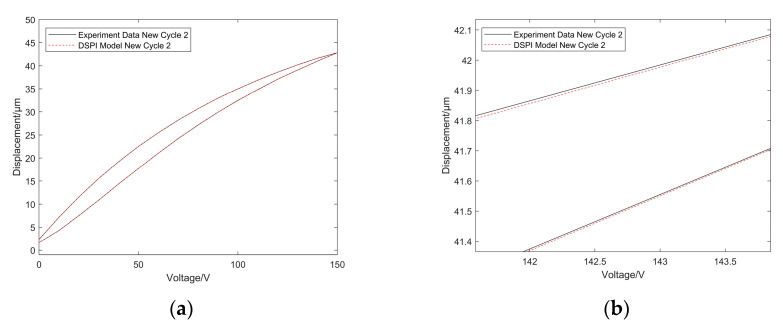
DSPI model: (**a**) modeling of the new second cycle data hysteresis characteristics; (**b**) local enlargement.

As shown in Figure 13b, there are two separation points: Cycle 2 Separation Point 1 and Cycle 2 Separation Point 2, which separate the data into three parts. The one-sided play operator is also selected conditionally for each part of the data. The data of each part of the new second cycle separation was refitted, as shown in Figure 16a. It is worth noting that the DSPI model demonstrated excellent articulation at the separation points, resulting in a well-fitted overall model. A partial enlargement of the hysteresis characteristics of the new second cycle data can be seen in Figure 16b.

**Figure 17:**
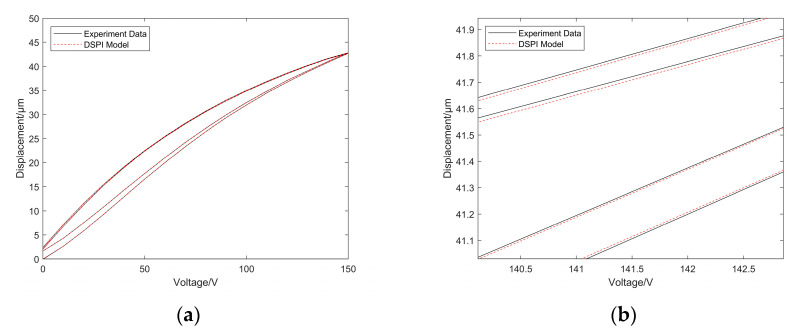
DSPI model: (**a**) modeling of the hysteresis characteristics after merging the first and new second cycle data; (**b**) local enlargement.

As shown in Figure 17, the hysteresis characteristics are modeled after combining the first and new second cycle data after modeling them separately. The overall modeling accuracy of the DSPI model is quite high, especially during startup. The hysteresis characteristics are also well captured by the model, providing an accurate representation of its behavior.

### 5.2. Results of the DSPI Inverse Model

**Figure 18:**
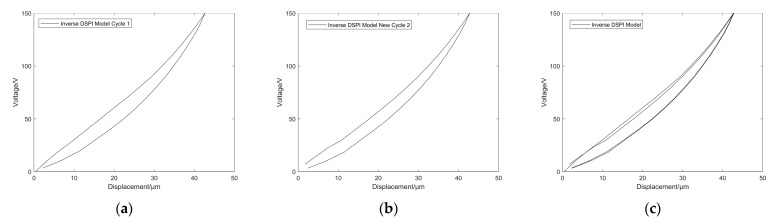
DSPI inverse model: (**a**) the first cycle; (**b**) the new second cycle; (**c**) overall modeling of the DSPI inverse model.

Figure 18a–c show the corresponding DSPI inverse models of Figure 15a, Figure 16a and Figure 17a respectively. The compensation voltage obtained from the DSPI inverse model is used for feedforward control. The experimental verification of the feedforward control compensation results is discussed and analyzed in the next Section 5.3.

### 5.3. Model Comparison

The results of the experimental compensation depend on the modeling results. The actual hysteresis curve error is obtained by observing the modeling results of different models with experiments. The effect of the control accuracy of the DSPI model can be visualized from this.

**Figure 19:**
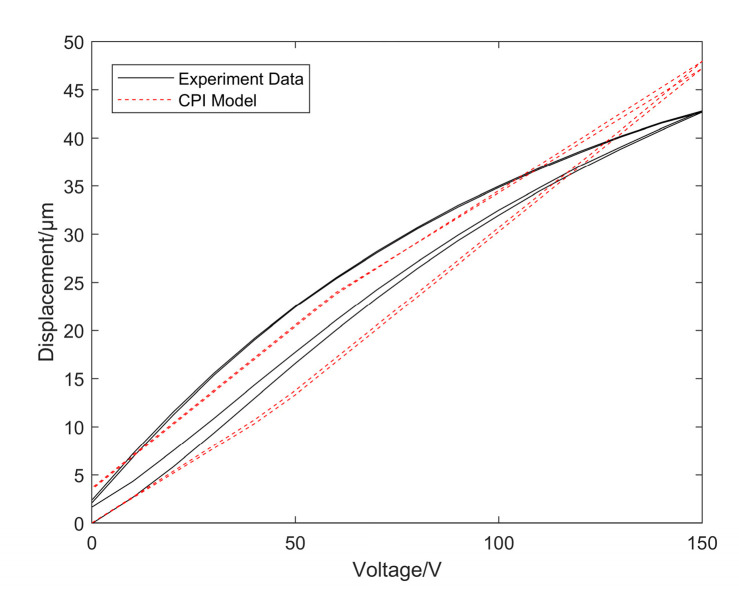
Hysteresis feature modeling of CPI model.

The DSPI model hysteresis characteristics were modeled, as shown in Figure 19. The CPI model hysteresis characteristics were modeled, as shown in Figure 19. By comparing the modeling plots shown in Figure 19 and Figure 17a, it can be seen that the DSPI model has higher modeling accuracy. The higher modeling accuracy at the start-up error can describe the nonlinear start-up error characteristic curve better under the open-loop control of the piezoelectric drive.

**Figure 20:**
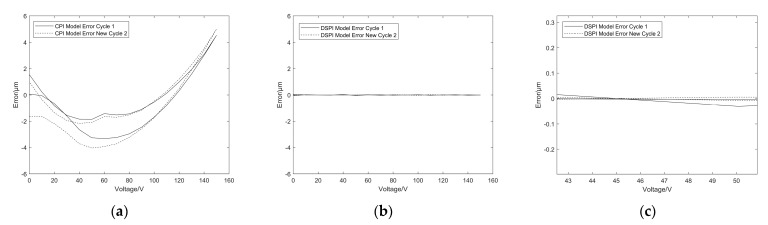
Modeling errors: (**a**) CPI model; (**b**) DSPI model; (**c**) local enlargement of (**b**).

As Figure 20 shows the modeling error of the CPI model and the modeling error of the DSPI model. Specifically, Figure 20a shows that the error curve of the CPI model is characterized by high fluctuations, resulting in a large modeling error. As shown in Figure 20b, the error curve of the DSPI model fluctuates very little, there is almost no fluctuation overall, and the modeling error is very small. The DSPI model has only a small defect at the 0 V description, and the modeling error is larger compared to the other voltages.

**Figure 21:**
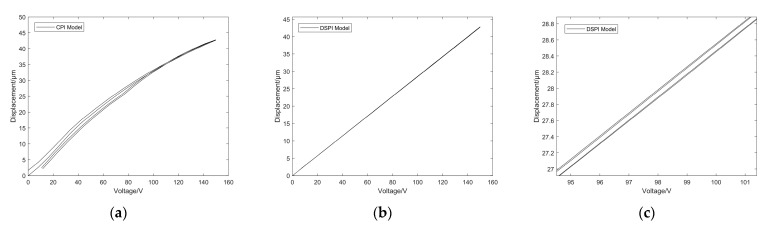
Comparison of the compensation control effect between the DSPI and CPI inverse models: (**a**) CPI inverse model; (**b**) DSPI inverse model; (**c**) local enlargement of (**b**).

In order to verify the compensating control effect of the DSPI inverse model, the following experiments were conducted on the experimental system in Figure 2 using the inverse model as input:

1. Adjust the laser interferometer. Connect the computer, laser interferometer, and controller. The relevant software is opened and waiting for a measurement;
2. Using the software to make the controller CPI inverse model loaded with the control voltage. The experimental voltage obtained by the CPI inverse model was used for experiment one, and the displacement was measured and recorded at equal time intervals using a laser interferometer. The experimental results are shown in Figure 21;
3. Experiment 2 is performed according to the voltage obtained from the DSPI inverse model, and the displacement is measured and recorded at equal time intervals using a laser interferometer. The time interval described in step 3 is kept the same as in 2;
4. Change the time interval to complete multiple measurements;
5. Check the apparatus and shut it down, and process the final experimental data.

Figure 21a shows the measured CPI inverse model compensation control effect. In contrast, Figure 21b shows the DSPI inverse model compensation control effect. In order to clearly show the hysteresis compensation effect, the plot uses a finer line style in order to present the error magnitude.

In the percentage index calculation results, the DSPI model improves the compensation control accuracy of the nonlinear start-up error characteristics of the piezoelectric actuator under open-loop control. It is experimentally verified that the DSPI model not only has higher modeling accuracy than the CPI model but also performs better in the final compensation results. The result of the average absolute error using the CPI inverse model compensation control is 1.897 μm, and the result of the average absolute error of the DSPI inverse model compensation control is 0.011 μm; the positioning accuracy of the DSPI model was improved by 99.420%. For the start-up, first cycle experimental data, the average absolute error result using CPI inverse model compensation control is 1.683 μm, and the average absolute error result of DSPI inverse model compensation control is 0.013 μm; the positioning accuracy of the DSPI model was improved by 99.228%. For the second cycle of experimental data, the average absolute error result using the CPI inverse model compensation control is 2.110 μm, and the average absolute error result of the DSPI inverse model compensation control is 0.009 μm; the positioning accuracy of the DSPI model was improved by 99.573%. The experiments verified the effectiveness of the DSPI model.

### 5.4. Another Experimental Comparison

**Figure 22:**
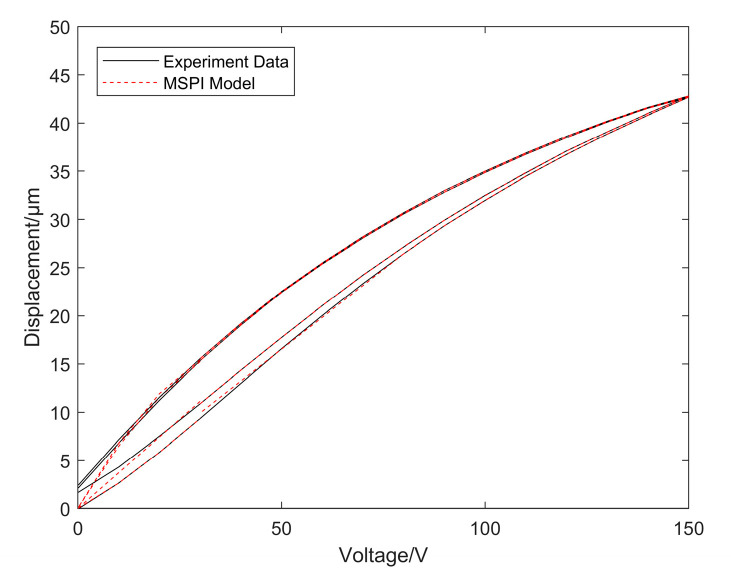
Hysteresis feature modeling of MSPI model.

In order to better verify the compensating control effect of the DSPI inverse model in this paper, the DSPI model was compared with the marked segmented Prandtle-Ishlinskii (MSPI) model [36]. The MSPI model is segmented by identifying the hysteresis tangent slope marker points, and the MSPI model can further improve the fitting accuracy of the hysteresis nonlinearity through the joint fitting of each segment. This modeling approach is good, but it does not take into account the effect of start-up errors. The experimental results are shown in Figure 22 below.

The hysteresis characteristics of the MSPI model are shown in Figure 22. By comparing the modeling plots shown in Figure 22 and Figure 17a, it can be observed that the DSPI model has higher modeling accuracy than the MSPI model. In particular, the modeling error of the MSPI model is large at the start-up end and at the finish end, meaning the nonlinear start-up error characteristic curve under the open-loop control of the piezoelectric actuator cannot be described well. DSPI model modeling performs better at the start and the finish, and the hysteresis characteristics can be depicted with high accuracy.

**Figure 23:**
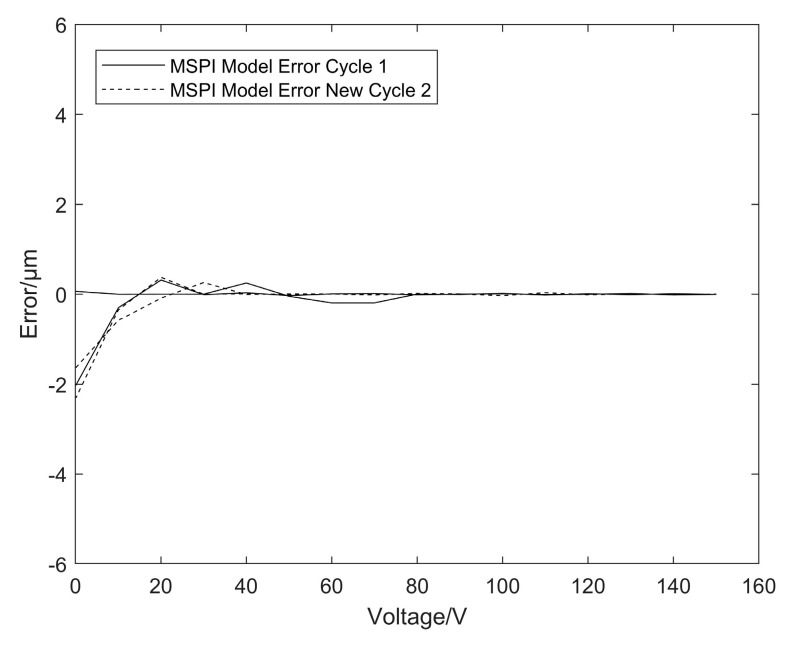
Modeling error of MSPI model.

The modeling error of the MSPI model is shown in Figure 23, from which it can be seen that the error curve of the MSPI model fluctuates greatly around 0 V, and the modeling error is relatively large. By comparing Figure 20b and Figure 23, it can be seen that the DSPI model has much smaller errors, and its error curve fluctuates less compared to the MSPI model.

**Figure 24:**
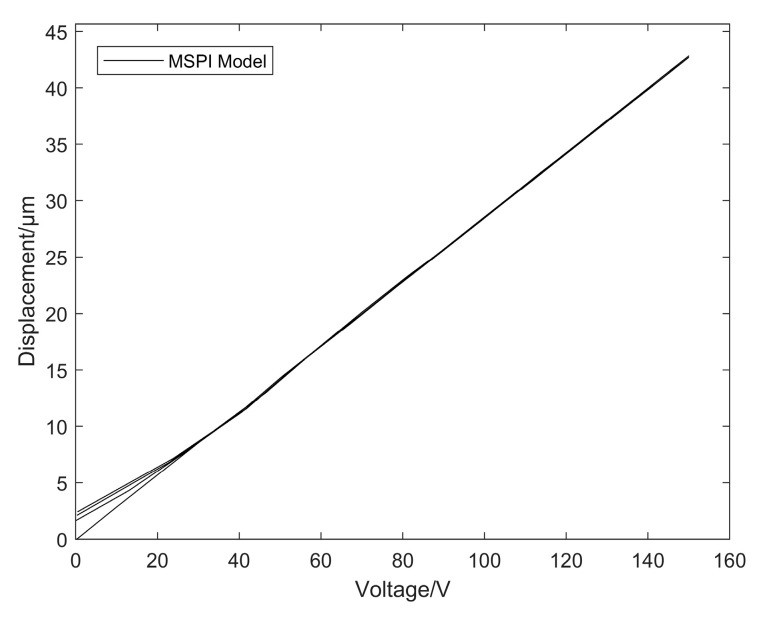
Compensating control effect of MSPI inverse model.

Figure 24 shows the MSPI inverse model compensation control effect measured experimentally. In contrast, Figure 23 shows the DSPI inverse model compensation control effect. As shown in Figure 24, the MSPI inverse model compensation control is poor near 0 V. The DSPI inverse model compensation performs significantly better. In the percentage index calculation results, the average absolute error result of the MSPI inverse model compensation control is 0.152 μm, and the average absolute error result of the DSPI inverse model compensation control is 0.011 μm; the positioning accuracy of the DSPI model was improved by 92.763%.

**Figure 25:**
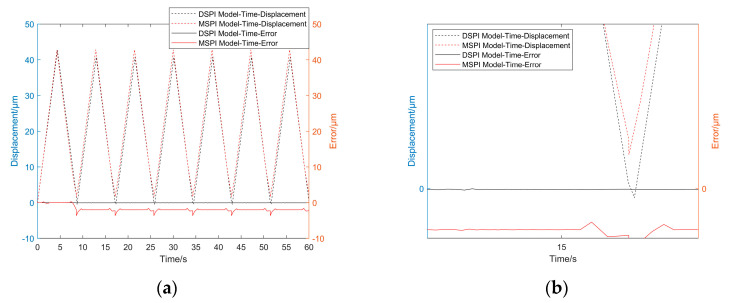
Time–displacement–error curves of DSPI Model and MSPI Model: (**a**) overall diagram; (**b**) local enlargement.

The figure displayed in Figure 25 shows the time–displacement–error curves for both the DSPI and MSPI models. The horizontal axis is time, the left vertical axis is displacement, and the right vertical axis is the error value of the desired displacement minus the displacement after compensation by the DSPI model and MSPI model. Since the MDPI model does not take into account the effect of start-up errors, the error values are accumulating from the second cycle onwards as time passes. The DSPI model takes into account the effect of the start-up error, which is small and always fluctuates around 0. This validates that the DSPI model can well describe the nonlinear start-up error characteristic curve of the piezoelectric actuator under open-loop control again.

## 6. Conclusions

The DSPI model effectively solves the problem of the low compensation accuracy of the nonlinear start-up error characteristics of the piezoelectric actuator under open-loop control. Experiments proved that the DSPI model not only has a higher modeling accuracy than the CPI model but also has better performance in terms of compensation results. The DSPI model improves the positioning accuracy by 99.427% compared to the traditional PI model. The DSPI model improves the localization accuracy by 92.763% when compared to the MSPI model. Although many scholars at home and abroad have adopted various methods to compensate for and control the nonlinear characteristics of piezoelectric actuators, they have not taken into account the effect of starting errors on the hysteresis characteristics. This paper analyzes the causes of starting errors in terms of the physical properties of the material and the magnitude of the voltage: starting errors are caused by the material properties of the piezoelectric ceramic, and the magnitude of the voltage determines the magnitude of the starting errors. Based on the analysis of the start-up error characteristic 1: “When the given linear voltage drops from 150 V to 0 V, the displacement value of the start-up first cycle data is not 0, and it does not return to the origin, which will generate the start-up error”. Start-up error characteristic 3 was found in the process of modeling the DSPI model. That is, the error of the experimental data from the second lap is very small, and it can be neglected, only considering the impact of the first cycle starting error on the experimental data. During the analysis of the causes of the starting error, starting error characteristic 2 was also found: the starting error is also influenced by the voltage. When the given voltage becomes larger, the starting error will become larger; when the given voltage becomes smaller, the starting error will become smaller.

The DSPI model hysteresis compensation method takes into account the influence of the start-up error value on the modeling, the characteristic hysteresis parameters, which are based on experimental data, and the model construction, which is simple with various advantages. In this paper, the PI model is still used as the basis to solve the compensation control problem. This is a continuation of the previous work. The research progress in this paper provides a modeling idea and theoretical basis for solving the problem of low compensation accuracy of nonlinear start-up error characteristics under the open-loop control of a piezoelectric actuator.

## Figures and Tables

**Table 1:** Start-up error values at different given voltages.

The Given Voltage	Start-Up Error Value
0–150–0 V	2.117105 μm
0–100–0 V	1.411403 μm
0–50–0 V	0.827996 μm

## Data Availability

The datasets during the current study are available from the corresponding author upon reasonable request.
